# Mucin1 shifts Smad3 signaling from the tumor-suppressive pSmad3C/p21^WAF1^ pathway to the oncogenic pSmad3L/c-Myc pathway by activating JNK in human hepatocellular carcinoma cells

**DOI:** 10.18632/oncotarget.2973

**Published:** 2015-01-20

**Authors:** Qiongshu Li, Guomu Liu, Hongyan Yuan, Juan Wang, Yingying Guo, Tanxiu Chen, Ruiping Zhai, Dan Shao, Weihua Ni, Guixiang Tai

**Affiliations:** ^1^ Department of Immunology, College of Basic Medical Science, Jilin University, Changchun 130021, China

**Keywords:** MUC1, pSmad3L, pSmad3C, HCC, JNK

## Abstract

Mucin1 (MUC1) is a transmembrane glycoprotein that acts as an oncogene in human hepatic tumorigenesis. Hepatocellular carcinoma (HCC) cells often gain advantage by reducing the tumor-suppressive activity of transforming growth factor beta (TGF-β) together with stimulation of its oncogenic activity as in MUC1 expressing HCC cells; however, molecular mechanisms remain largely unknown. Type I TGF-β receptor (TβRI) and c-Jun NH2-terminal kinase (JNK) differentially phosphorylate Smad3 mediator to create 2 phosphorylated forms: COOH-terminally phosphorylated Smad3 (pSmad3C) and linker-phosphorylated Smad3 (pSmad3L). Here, we report that MUC1 overexpression in HCC cell lines suppresses TβRI-mediated pSmad3C signaling which involves growth inhibition by up-regulating p21^WAF1^. Instead, MUC1 directly activates JNK to stimulate oncogenic pSmad3L signaling, which fosters cell proliferation by up-regulating c-Myc. Conversely, MUC1 gene silencing in MUC1 expressing HCC cells results in preserved tumor-suppressive function via pSmad3C, while eliminating pSmad3L-mediated oncogenic activity both *in vitro* and *in vivo*. In addition, high correlation between MUC1 and pSmad3L/c-Myc but not pSmad3C/p21^WAF1^ expression was observed in HCC tissues from patients. Collectively, these results indicate that MUC1 shifts Smad3 signaling from a tumor-suppressive pSmad3C/p21^WAF1^ to an oncogenic pSmad3L/c-Myc pathway by directly activating JNK in HCC cells, suggesting that MUC1 is an important target for HCC therapy.

## INTRODUCTION

Mucin1 (MUC1) is a transmembrane glycoprotein that plays a key role as an oncogene in the tumorigenesis of many human adenocarcinomas [1–4]. The extracellular domain of MUC1 contains a variable number of tandem repeat (VNTR) regions composed of 20–200 tandem repeat (TR) units of 20 amino acids [5]. The cytoplasmic domain (MUC1-CD) plays a role in many signaling pathways that regulate cell survival, proliferation and apoptosis, including those involving Wnt/β-catenin [6], phosphatidylinositol-3-kinase (PI3K)/protein kinase B (AKT) [4], c-Src [7], growth factor receptor-bound protein 2 (Grb2)/son of sevenless (Sos) [8], p53 [9], glycogen synthase kinase 3β (GSK3β) [6], epidermal growth factor receptor (EGFR) [10, 11], and nuclear factor-κB (NF-κB) [12, 13]. Recently, studies have shown that MUC1 is overexpressed in human hepatocellular carcinoma (HCC) tissue and cell lines [14–16]. However, the role of MUC1 in HCC progression remains unclear. In a recent study, we showed that MUC1 gene silencing decreased the malignancy of the SMMC-7721 HCC cell line [17], suggesting that MUC1 plays a key role in HCC tumorigenesis. What is particularly attractive is the observation that MUC1 gene silencing decreased the Smad3 mRNA level based on a global gene expression analysis, suggesting a new mechanism in which MUC1 mediates transforming growth factor beta (TGF-β) signaling.

TGF-β signaling plays a tumor suppressive role in normal epithelia by inhibiting cell proliferation and inducing apoptosis but accelerates the progression of established cancers through autocrine and paracrine mechanisms [18–22]. However, the mechanisms underlying this dual role of TGF-β signaling are still unclear. TGF-β binds to type I and type II receptor serine/threonine kinase on the cell surface, activating Smads to regulate gene expression, which is a classical pathway for TGF-β signaling transduced from the cell membrane to the nucleus. Smad3 consists of three domains: (1) an N-terminal Mad-homology 1 (MH1) domain that carries nuclear localization signals (NLSs) and a DNA-binding domain; (2) a middle linker domain that is rich in prolines and phosphorylatable serines or threonines such as residues Ser-213, Ser-204, Ser-208 and Thr-179; and (3) a C-terminal MH2 domain that contains a Ser-Ser-X-Ser motif [23].

Membrane-bound and cytoplasmic protein kinases differentially phosphorylate Smad3 to create C-tail (C) or the linker (L) phosphorylated (p, phospho-) isoforms. According to domain-specific phosphorylation, distinct transcriptional responses, and selective metabolism, Smad phospho-isoform pathways can be grouped into 2 types: COOH-terminally phosphorylated Smad3 (pSmad3C) and linker-phosphorylated Smad3 (pSmad3L). Recently, the studies by Sekimoto *et al* and Matsuzaki *et al* have shown that TGF-β type I receptor (TβRI) phosphorylates Smad3 at the COOH-terminal (pSmad3C) thus inhibiting cell proliferation by up-regulating p21^WAF1^ in human gastric mucosa epithelial cells (RGM1) and human intestinal epithelial cells; the activated c-Jun N-terminal kinase (JNK) phosphorylates Smad3 at the Linker-terminal Ser-213 site (pSmad3L) to promote cell proliferation and carcinogenesis by up-regulating c-Myc in Ras-transformed human RGM1 cells and colon cancer cells [24, 25]. Further studies demonstrate that pSmad3L inhibits pSmad3C resulting in the suppression of cell cycle blockade; thus, the pSmad3L/c-Myc pathway and the pSmad3C/p21^WAF1^ pathway are reversible and antagonistic. These results described above suggest a novel theory that the dual roles of TGF-β signaling depend on Smad3 phospho-isoforms. Clinical observations also support the roles for pSmad3L (Ser-213) as a tumor promoter and pSmad3C as a tumor suppressor in virus infection-related HCC tissues [26]. However, the molecular mechanisms underlying how Smad3 signaling shifts from tumor-suppression to oncogenesis have not been well characterized. Several studies reported that inflammatory cytokines activate JNK, resulting in the switch in Smad3 signaling from tumor-suppression to oncogenesis [27–30]. The latest study by Nagata *et al* showed that the inhibition of JNK shifts Smad3 signaling from oncogenesis to tumor-suppression in DEN induced rat hepatocellular carcinoma [31], suggesting that JNK is a key conductor in the switch in Smad3 signaling. However, it is not known what activates JNK and then shifts the Smad3 signaling.

Our previous study found that MUC1 gene silencing decreased Smad3 mRNA level in HCC cells [17], and another study has shown that MUC1 can active JNK to inhibit cell apoptosis [32], thus leading to the hypothesis that MUC1 shifts Smad3 from tumor-suppression to oncogenesis by activating JNK in HCC cells. In this study, to investigate the effects of MUC1 on cell proliferation and the dual role of Smad3 signaling in HCC cells, we established MUC1 gene silencing and overexpressing cell lines, and then investigated the relationship between MUC1 and JNK activation *in vitro* and *in vivo*. The present study indicates a novel mechanism by which MUC1 promotes cancer progression and provides an attractive target for HCC therapy.

## RESULTS

### MUC1 expression enhances HCC cell proliferation

**Figure 1:**
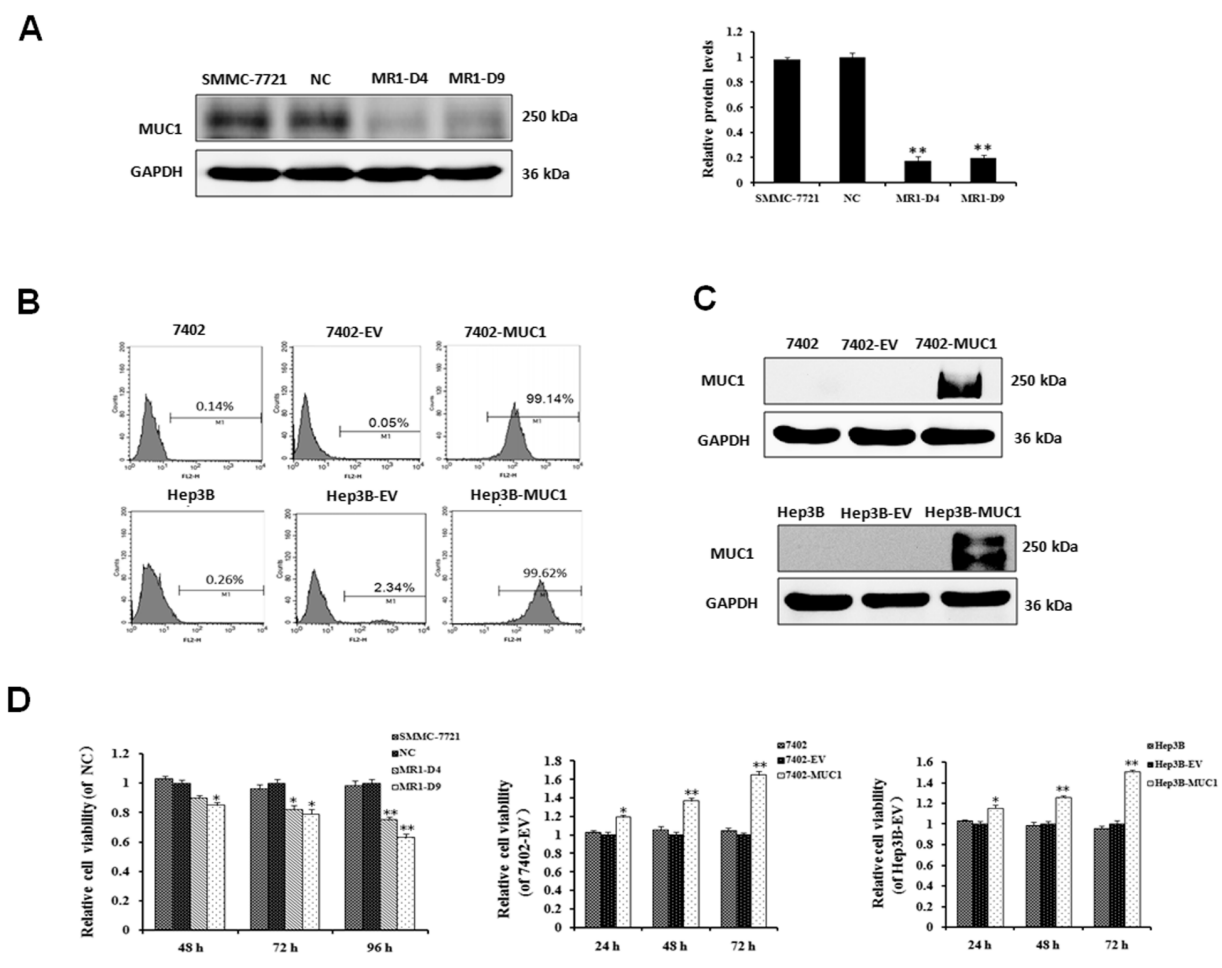
MUC1 expression enhances HCC cell proliferation **(A)** Knockdown of MUC1 by siRNA in SMMC-7721 cells. Western blotting analysis for MUC1 expression after normalization to GAPDH and quantification. The data are expressed as the means ± SD of three independent experiments. **P < 0.01 compared with the control. **(B and C)** Overexpression of MUC1 by infection with lentivirus construct insertion of the full-length human MUC1 gene in Bel7402 and Hep3B HCC cell lines. MUC1 expression in Bel7402, 7402-EV, 7402-MUC1, Hep3B, Hep3B-EV and Hep3B-MUC1 cells were analyzed by flow cytometry using the anti-MUC1 primary antibody (GP1.4) and PE-conjugated secondary antibody (B), and Western blotting (C) with GAPDH was used as a loading control. **(D)** Cell viability was determined using the WST-1 assay. Triplicate wells containing 5 × 10^3^ cells were used to evaluate cell viability at 24 h intervals. The relative cell viability was calculated as the A450 nm (MUC1-silenced or overexpressed cells at Tn)/A450 nm (control at Tn) × 100%. The data are expressed as the means ± SD of three independent experiments. *P < 0.05, **P < 0.01 compared with the control.

To determine the effect of MUC1 expression in HCC, the MUC1 gene was silenced in SMMC-7721 cells using RNAi as previously described [17]. Two independent MUC1 knockdown stable cell clones, named MR1-D4 and MR1-D9, and a negative control clone, named NC, were analyzed by Western blotting (Figure 1A). The results showed that MUC1 expression in the MR1-D4 and MR1-D9 cell clones was significantly decreased compared to NC or SMMC-7721 cells (*P* < 0.01). The silencing efficiency in clones MR1-D4 and MR1-D9 reached 82.34% and 80.13%, respectively. Both Bel7402 and Hep3B cells, which almost do not express MUC1, were infected with a lentivirus construct inserting full-length human MUC1. Then, MUC1 expression was identified by flow cytometry and Western blotting (Figure 1B and 1C). Two MUC1-overexpressing cell lines, 7402-MUC1 and Hep3B-MUC1, and two negative controls, named 7402-EV and Hep3B-EV, were established. The results showed that MUC1 expression efficiency in 7402-MUC1 and Hep3B-MUC1 cell lines reached 99.14% and 99.62%, respectively. We used these MUC1 gene silencing and overexpressing HCC cell lines for the subsequent studies.

To investigate the influence of MUC1 on HCC cell proliferation, we performed a WST-1 cell viability assay. The result showed that the cell viability of MUC1-knockdown MR1-D4 and MR1-D9 cells was significantly reduced compared to NC or SMMC-7721 cells. In contrast, MUC1 overexpression in Bel7402 and Hep3B cells increased the cell viability compared to the control cells (Figure 1D). The result demonstrates that MUC1 enhances HCC cell proliferation.

### MUC1 shifts Smad3 signaling from a tumor-suppressive pSmad3C/p21^WAF1^ pathway to an oncogenic pSmad3L/c-Myc pathway in HCC cells

**Figure 2:**
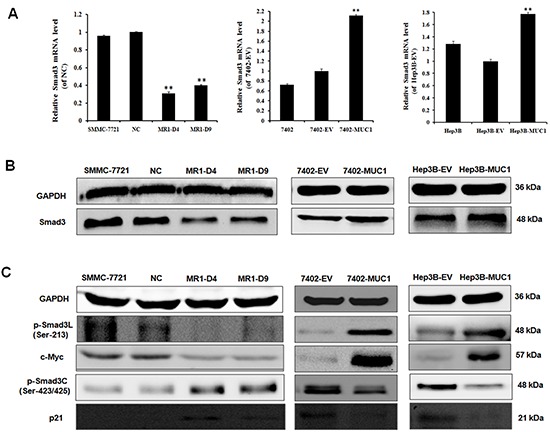
MUC1 shifts Smad3 signaling from a tumor-suppressive pSmad3C/p21^WAF1^ pathway to an oncogenic pSmad3L/c-Myc pathway in HCC cells **(A)** The mRNA level of Smad3 in MUC1 knockdown and overexpression HCC cell lines was detected by qRT-PCR. Bars represent the relative mRNA level when compared to the control cells. The data are expressed as the means ± SD of three independent experiments. **P < 0.01 compared with the control. **(B and C)** Cell lysates of MUC1 knockdown and overexpression HCC cell lines were analyzed by Western blotting for the expression of Smad3, p-Smad3L (Ser-213), c-Myc, p-Smad3C (Ser-423/425) and p21^WAF1^. The results are representative of three independent experiments.

Our previous study showed that MUC1 gene silencing decreased the malignancy and Smad3 mRNA level in HCC cells [17]; to investigate the influence of MUC1 on Smad3 expression in HCC cells, Smad3 mRNA and protein levels were determined by qRT-PCR and Western blotting. The results showed that Smad3 levels in MUC1 knocked down MR1-D4 and MR1-D9 cells were significantly reduced but were increased in MUC1 overexpressing Bel7402 and Hep3B cells compared to the control cells (Figure 2A and 2B), suggesting that MUC1 can affect Smad3 signaling in HCC cells. To investigate whether MUC1 regulates Smad3 signaling, Smad3 phospho-isoforms and the corresponding target gene expression were detected by Western blotting. The results showed that the levels of phosphorylated Smad3L (Ser-213) and its target gene c-Myc were significantly decreased, but the levels of phosphorylated Smad3C (Ser-423/425) and its target gene p21^WAF1^ were significantly elevated in the MUC1 knocked down MR1-D4 and MR1-D9 cells compared to the NC or SMMC-7721 cells. In contrast, pSmad3L (Ser-213) and the c-Myc expression were upregulated, but pSmad3C (Ser-423/425) and p21^WAF1^ expression were downregulated in MUC1-overexpressing Bel7402 and Hep3B cells compared to the control cells (Figure 2C). These results indicate that MUC1 shifts Smad3 signaling from a tumor-suppressive pSmad3C/p21^WAF1^ pathway to an oncogenic pSmad3L/c-Myc pathway.

### MUC1 modulates Smad3 signaling by directly binding and activating JNK in HCC cells

**Figure 3:**
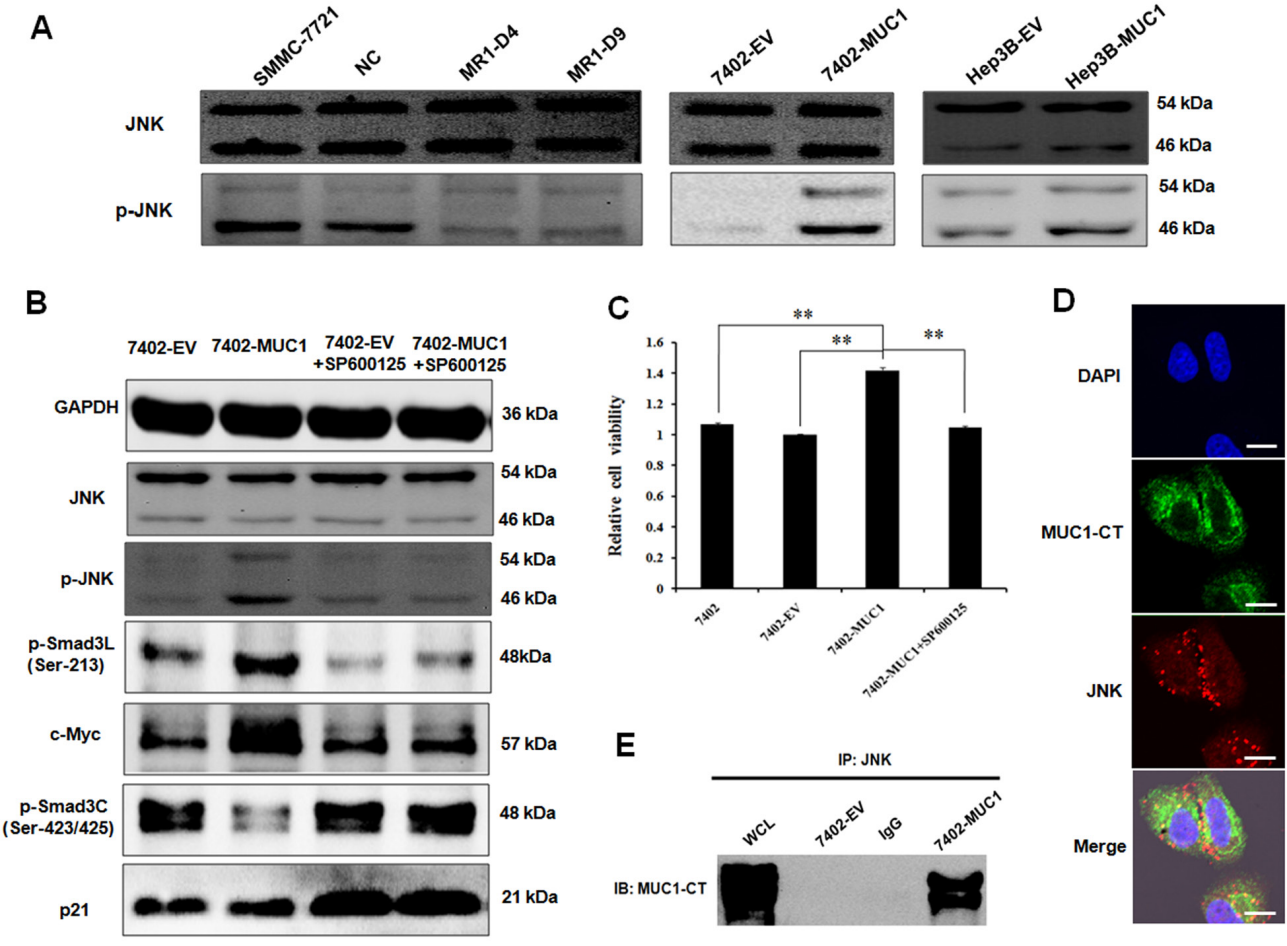
MUC1 modulates Smad3 signaling by directly binding and activating JNK in HCC cells **(A)** Cell lysates of MUC1 knockdown and overexpression HCC cell lines were analyzed by Western blotting for the expression of JNK and p-JNK. **(B)** Cell lysates of 7402-EV and 7402-MUC1 cells treated with or without JNK inhibitor SP600125 (30 μM) were analyzed by Western blotting for the expression of JNK, p-JNK, p-Smad3L (Ser-213), c-Myc, p-Smad3C (Ser-423/425) and p21^WAF1^, GAPDH was used as a loading control. **(C)** Bel7402, 7402-EV and 7402-MUC1 cells were treated with JNK inhibitor SP600125 (30 μM) for 48 h, and cell viability was determined by the WST-1 assay. The data are expressed as the means ± SD of three independent experiments. **P < 0.01 compared with the control. **(D)** 7402-MUC1 cells were detected by immunofluorescence staining with the hamster anti-MUC1-CT and mouse anti-JNK primary antibodies and FITC-conjugated goat anti-hamster (green) and PE-conjugated goat anti-mouse (red) secondary antibodies. The nuclei were stained with DAPI (blue), and the cells were observed by confocal laser scanning microscopy (Olympus FV1000). The merged image indicates the overlap of MUC1-CT (green), JNK (red) and DAPI (blue), and yellow in the merged image indicates overlap of green and red labels. The scale bar indicates 10 μm. **(E)** Cell lysates from the 7402-EV and 7402-MUC1 cells were subjected to immunoprecipitation (IP) with anti-JNK antibody or normal IgG and then immunoblotted (IB) with anti-MUC1-CT antibody. WCL indicates the whole cell lysate that was not subjected to immunoprecipitation.

Because JNK is a key conductor in the switch in Smad3 signaling, to clarify the mechanism by which MUC1 shifts Smad3 signaling, the effect of MUC1 on JNK activation was detected by Western blotting analysis. The results showed that the level of phosphorylated JNK was significantly decreased in the MUC1-knockdown MR1-D4 and MR1-D9 cells compared to the NC or SMMC-7721 cells; in contrast, JNK was obviously activated in the MUC1-overexpressing Bel7402 and Hep3B cells compared to the control cells (Figure 3A). Moreover, when the JNK inhibitor, SP600125, was administered, cell viability was inhibited, and the levels of pSmad3L (Ser-213) and c-Myc were decreased, whereas the levels of pSmad3C (Ser-423/425) and p21^WAF1^ were elevated (Figure 3B and 3C). These results indicate that MUC1 activates JNK and enhances cell proliferation by shifting Smad3 signaling from a tumor-suppressive pSmad3C/p21^WAF1^ pathway to an oncogenic pSmad3L/c-Myc pathway in HCC cells.

To thoroughly investigate the effect of MUC1 on JNK, MUC1-overexpressing 7402 cells were stained by hamster anti-MUC1-CT and mouse anti-JNK primary antibody, and FITC-conjugated goat anti-hamster and PE-conjugated goat anti-mouse secondary antibody. Some overlying yellow fluorescence was observed with confocal fluorescence microscopy (Figure 3D). Further, cell lysates were immunoprecipitated using anti-JNK antibody followed by immunoblot analysis using anti-MUC1-CT antibody; MUC1-CT was detected and directly bound to JNK in 7402-MUC1 cells but not in the 7402-EV cells or the controls (Figure 3E). These results indicate that MUC1 directly binds and activates JNK in HCC cells.

### TGF-β has no effect on the MUC1-induced switch in Smad3 signaling in HCC cells

**Figure 4:**
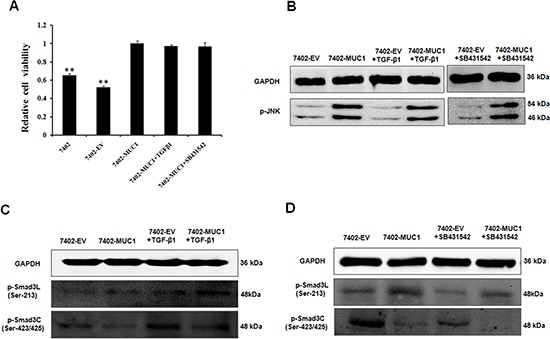
TGF-β has no effect on the MUC1-induced switch in Smad3 signaling in HCC cells **(A)** Bel7402, 7402-EV and 7402-MUC1 cells were serum-starved for 16 h before treatment with exogenous TGF-β1 (50 pM) and the TβR inhibitor SB431542 (20 μM) for 48 h, and cell viability was determined using the WST-1 assay. The data are expressed as the means ± SD of three independent experiments. **P < 0.01 compared with the 7402-MUC1 cells. **(B, C** and **D)** The 7402-EV and 7402-MUC1 cells were serum-starved for 16 h before treatment with or without exogenous TGF-β1 (50 pM) and TβR inhibitor SB431542 (20 μM), and analysis by Western blotting for the expression of p-JNK (B), p-Smad3L (Ser-213) and p-Smad3C (Ser-423/425) (C and D). GAPDH was used as the loading control. The results are representative of three independent experiments.

Studies have shown that TGF-β can activate JNK by mediating Smad-independent signaling [33, 34], and our previous study showed that MUC1 expression induces TGF-β1 secretion (data not shown), leading to a hypothesis that MUC1-induced TGF-β secretion influences JNK activation and then mediates Smad3 signaling. Thus, the 7402-EV and 7402-MUC1 cells were treated with exogenous TGF-β1 and TGF-β receptor (TβR) inhibitor (SB431542). Then, the cell viability was analyzed by WST-1, and the Smad3 signaling molecules and JNK activation were detected by Western blotting. The effects of exogenous TGF-β1 and TβR inhibitor on cell proliferation and JNK activation were not observed in the MUC1-overexpressing cells (Figure 4A and 4B). Moreover, TGF-β1 enhanced the levels of both pSmad3L (Ser-213) and pSmad3C (Ser-423/425), which were inhibited by the TβR inhibitor in both 7402-EV and 7402-MUC1 cells (Figure 4C and 4D). These results indicate that JNK activation is dependent on MUC1 but not TGF-β, and TGF-β has no effect on MUC1-induced switch in Smad3 signaling in HCC cells.

### Knockdown of MUC1 suppresses tumor growth and JNK/pSmad3L/c-Myc pathway in mice

**Figure 5:**
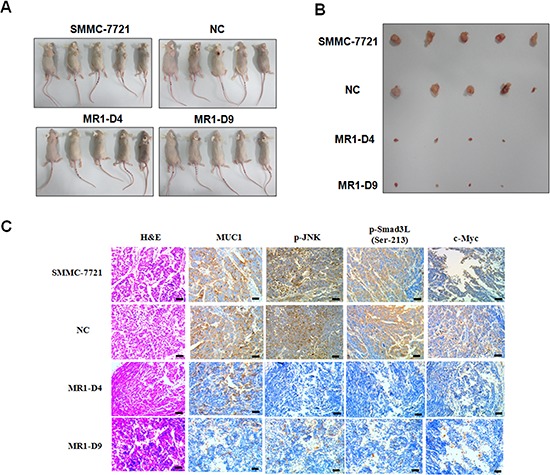
Knockdown of MUC1 suppresses tumor growth and JNK/pSmad3L/c-Myc pathway in mice **(A)** BALB/c nude mice subcutaneous transplant tumor model was established using SMMC-7721, NC, MR1-D4 and MR1-D9 cells. **(B)** Tumors in mice were dissected and photographed on day 21 post-injection. The images were captured showing tumor sizes for each group. **(C)** Tumors from mice were detected for the expression of MUC1, p-JNK, p-Smad3L (Ser-213) and c-Myc by immunohistochemical staining. Sections were examined on an inverted fluorescence microscope (IX71; Olympus) at 100 × magnification. The scale bar indicates 50 μm.

To evaluate the effect of MUC1 on Smad3 signaling *in vivo*, SMMC-7721, NC, MR1-D4 and MR1-D9 cells were inoculated subcutaneously into BALB/c nude mice to establish a subcutaneous transplant tumor model. Twenty-one days after tumor implantation, the mice were killed, and the tumors were dissected. As shown in Figure 5A and 5B, the MR1-D4 and MR1-D9 groups grew smaller tumors compared with the SMMC-7721 or NC groups. This result indicates that MUC1 plays an important role in HCC tumorigenesis. Immunohistochemical staining showed that MUC1, p-JNK, pSmad3L and c-Myc expression were highly positive in tumor tissues from the SMMC-7721 or NC groups but were weakly positive or even negative in tumor tissues from the MR1-D4 and MR1-D9 groups (Figure 5C). These results indicate a correlation between MUC1 and the JNK/pSmad3L/c-Myc pathway expression *in mice*.

### The expression of MUC1 is consistent with that of the pSmad3L/c-Myc pathway but inverse to that of the pSmad3C/p21^WAF1^ pathway in tumor tissues from HCC patients

**Figure 6:**
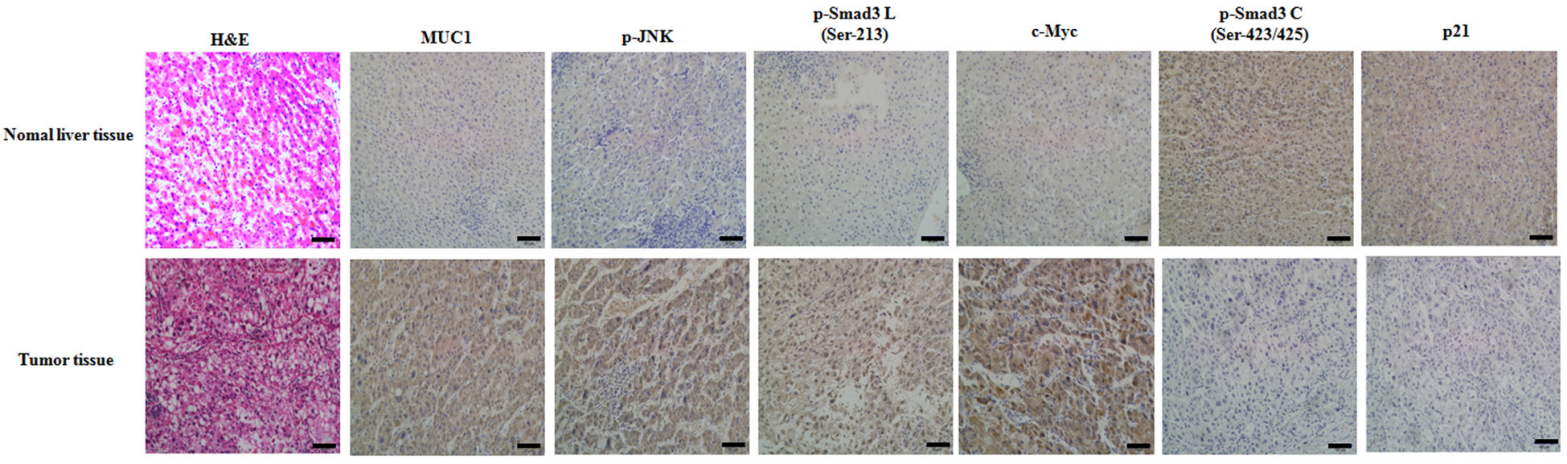
The expression of MUC1 is consistent with that of the pSmad3L/c-Myc pathway but inverse to that of the pSmad3C/p21^WAF1^ pathway in tumor tissues from HCC patients The expression of MUC1, p-JNK, pSmad3L(Ser-213), c-Myc, pSmad3C (Ser-423/425) and p21^WAF1^ in normal liver tissues from hemangioma patients and tumor tissues from HCC patients were investigated using immunohistochemical staining. The sections were examined on an inverted fluorescence microscope (IX71; Olympus) at 100 × magnification. The scale bar indicates 50 μm.

To further investigate the association between MUC1 and the pSmad3L/c-Myc pathway or pSmad3C/p21^WAF1^ expression in human, normal liver tissues from hemangioma patients and tumor tissues from HCC patients were collected and analyzed preliminarily by immunohistochemical staining. Both MUC1 and p-JNK/pSmad3L/c-Myc expression were highly positive in tumor tissues but weakly positive in normal liver tissues (Figure 6). However, pSmad3C/p21^WAF1^ expression was weakly positive in tumor tissues but highly positive in normal liver tissues. The result indicates a high positive correlation between MUC1 and pSmad3L/c-Myc expression, and a reverse correlation between MUC1 and pSmad3C/p21^WAF1^ expression in HCC patients.

## DISCUSSION

Our previous study found that MUC1 gene silencing decreased HCC cell proliferation and the Smad3 signaling pathway based on global gene expression analysis [17], suggesting a new mechanism that MUC1 may mediate Smad3 signaling. Smad3 as a central mediator for the classical pathway of TGF-β signaling mainly transmits tumor-suppressive signaling. Recently, Matsuzaki *et al* demonstrated that the roles of TGF-β signaling are dependent on Smad3 phospho-isoforms in tumor progression. Activated TβRI and JNK differentially phosphorylates the mediator Smad3 to become pSmad3C (Ser-423/425) and pSmad3L (Ser-213) and then transmits tumor-suppressive or oncogenic TGF-β signaling, respectively, by mediating distinct transcriptional responses [24, 25]. Thus, we proposed the hypothesis that MUC1 enhances HCC cell proliferation by activating Smad3L (Ser-213) signaling. In this study, we established MUC1 gene silencing of SMMC-7721, and MUC1 overexpression in Bel7402 and Hep3B HCC cell lines, investigating the effects of MUC1 on cell proliferation and Smad3 signaling.

We performed WST-1 and Western blotting assays and found that MUC1 expression, cell viability and Smad3 expression changed consistently in response to MUC1 gene silencing and overexpression in HCC cell lines, suggesting that MUC1 may enhance HCC cell proliferation by mediating Smad3 signaling. Further study demonstrated that the expression of MUC1 is consistent with that of pSmad3L and c-Myc but inverse to that of pSmad3C and p21^WAF1^, suggesting that MUC1 shifted Smad3 signaling from tumor-suppression to oncogenesis in tumor cells. MUC1 acts as an oncogene to promote tumor formation and progression by regulating multiple proliferation-promoting signaling pathways [1, 4]; however, whether MUC1 mediates Smad3 signaling in cancer cells has not previously been reported. Our results reveal a new mechanism in which MUC1 promotes cancer progression by mediating Smad3 signaling. The studies by Matsuzaki *et al* and Yamagata H *et al* also demonstrated that Smad3 signaling is shifted from tumor-suppression to oncogenesis in HCC and colorectal cancer [26, 35]; however, the molecular mechanisms have not been well characterized. Nagata showed that the inhibition of JNK switches Smad3 signaling from oncogenic pSmad3L/c-Myc to tumor-suppressive pSmad3C/p21^WAF1^ in DEN-induced rat hepatocellular carcinoma [31]. In addition, some cytokines such as tumor necrosis factor-alpha (TNF-α), interleukin-1 beta (IL-1β) and in-terleukin-6 (IL-6) induced by chronic liver inflammation including hepatitis viral infection and steatohepatitis activate JNK, resulting in a switch in Smad3 signaling from tumor-suppression to oncogenesis [27–30]. These results suggest that JNK has been considered a key conductor in the switch in Smad3 signaling; however, the signal molecule upstream of JNK that shifts Smad3 signaling has not been thoroughly elucidated. To clarify whether MUC1 is the activator of JNK, we detected the effect of MUC1 on JNK activation and found that MUC1 expression activated JNK in HCC cells. Meanwhile, immunofluorescence confocal microscopy and co-immunoprecipitation analysis showed that MUC1 directly interacted with JNK in MUC1-overexpressing HCC cells, which is consistent with Chen *et al*, who showed that exogenous MUC1 interacted with JNK in HCT116 cells [32]. Moreover, pharmacological reduction of JNK activity not only shifted Smad3 signaling from oncogenesis to tumor-suppression but also inhibited cell proliferation in MUC1-overexpressing HCC cells. Because several studies by Sorrentino et al and Yamashita et al have shown that TGF-β activates JNK by mediating Smad-independent signaling [36, 37] and our previous study showed that MUC1 expression induces TGF-β1 secretion (data not shown) in HCC cells, we sought to investigate whether MUC1-induced TGF-β secretion could activate JNK and mediate Smad3 signaling. We treated 7402-EV and 7402-MUC1 cells with exogenous TGF-β1 and the TβR inhibitor and found that they had no effect on HCC cell proliferation, JNK activation and the switch in Smad3 signaling. These results suggested that JNK activation is dependent on MUC1 but not MUC1-induced TGF-β in HCC cells. Thus, these results suggest that MUC1 directly activates JNK, thus enhancing HCC cell proliferation by shifting Smad3 signaling from tumor-suppression to oncogenesis. Recently, Matsuzaki *et al* and Sekimoto *et al* also found that oncogenic Ras modulated Smad3 tumor-suppressive signaling by activating JNK *in vitro* and *in vivo* [24, 25, 38]; therefore, these results in combination with ours suggest that oncogenes play an important role in the switch in TGF-β signaling in tumor cells. TGF-β signaling has been considered a therapeutic target for many cancers, and our results demonstrate that the MUC1 oncogene, upstream of the TGF-β signaling is a more attractive therapeutic target for HCC.

To evaluate the effect of MUC1 on Smad3 signaling *in vivo*, the BALB/c nude mice subcutaneous transplant tumor model was established using SMMC-7721, NC, MR1-D4 and MR1-D9 cells; the results showed that MUC1 gene silencing inhibited tumor growth. Moreover, high correlation between MUC1 and pSmad3L/c-Myc but not pSmad3C/p21^WAF1^ expression was also observed in both tumors from nude mice and HCC tissues from patients, suggesting that MUC1 shifts Smad3 signaling from tumor-suppression to oncogenesis *in vivo*, which is consistent with clinical observations by Matsuzaki *et al*, which supports the roles for pSmad3L (Ser-213) as a tumor promoter and pSmad3C as a tumor suppressor in virus infection-related HCC tissues [25, 26]. These results suggest that MUC1 might be a more useful target and an independent prognostic factor for HCC.

**Figure 7:**
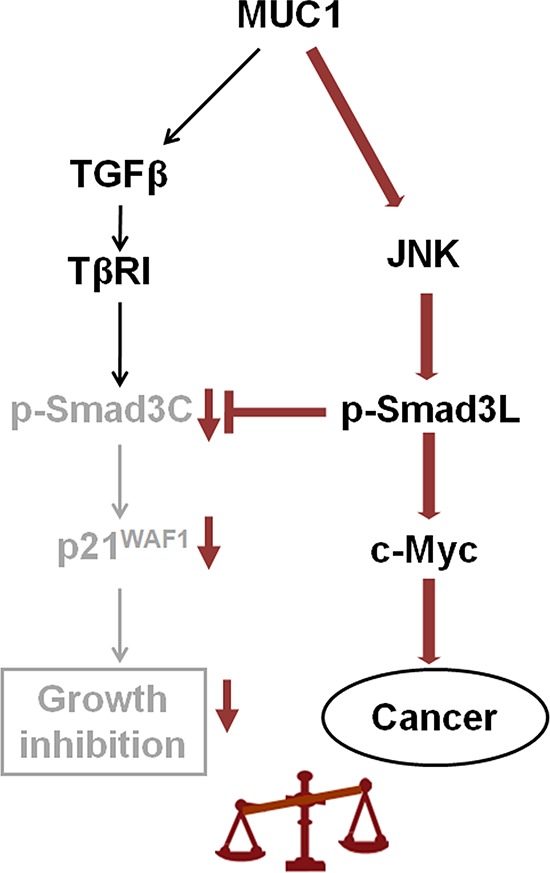
Proposed model how MUC1 promotes HCC cell proliferation by mediating Smad3 signaling Smad3-dependent signaling shows reversible switching between tumor suppressive TβRI/pSmad3C/p21^WAF1^ and oncogenic JNK/pSmad3L (Ser-213)/c-Myc signaling. MUC1 mediates the shutdown of pSmad3C-mediated signaling in HCC cells, while acquiring constitutively active JNK-mediated pSmad3L (Ser-213) signaling, thus participating in HCC cell proliferation by stimulating transcriptional activity of c-Myc gene and repressing that of p21^WAF1^.

Our present study showed that HCC cells affected by oncogene MUC1 undergo transition from the tumor-suppressive TβRI/pSmad3C/p21^WAF1^ pathway to the oncogenic JNK/pSmad3L/c-Myc pathway (Figure 7). Moreover, our previous study and another study by Wang SE *et al* demonstrated that oncogenes mediate autocrine TGF-β signaling [39], indicating that oncogenes not only alter intracellular signaling in cancer cells but also possess the proficiency to mediate the tumor microenvironment through TGF-β, and therefore they should be considered a dominant target for cancer therapy.

## MATERIALS AND METHODS

### Cell lines and lentiviral vectors

The HCC cell lines SMMC-7721, Bel7402 and Hep3B and the virus packaging cell line HEK293T were purchased from the Cell Bank of the Shanghai Institute of Cell Biology, Chinese Academy of Sciences. Cells were cultured in Iscove's modified Dulbecco's medium (IMDM) supplemented with 100 U/ml penicillin, 100 μg/ml streptomycin, and 10% fetal bovine serum (FBS) (Gibco-BRL, Carlsbad, CA, USA) in an incubator at 37°C and 5% CO_2_. The lentiviral vector pWPXLd and two packaging vectors, PAX2 and PMD2.G, were obtained from Addgene.

### Generation of stable MUC1 knockdown and overexpression in HCC cells

The MUC1 gene was silenced in SMMC-7721 cells using RNAi, as previously described [17]. Briefly, human MUC1 siRNA oligonucleotides were synthesized to target sequences of the MUC1 gene (GACTGATGCCAGTAGCACT), GenBank accession No. J05582. The siRNA was inserted into the expression vector pGCsilencer™ U6.Neo.GFP (Genechem Co., Ltd., Shanghai, China). A nonspecific siRNA was used as a negative control. The siRNA expression plasmids were transfected into SMMC-7721 cells using Lipofectamine 2000 (Invitrogen). Cells were screened with 1200 μg/ml G418 for three weeks to establish stable MUC1 knockdown cell lines. Two independent MUC1 knockdown clones (MR1-D4 and MR1-D9) and a negative control clone (NC) were isolated and identified by immunofluorescence and Western blotting. The stable MUC1 knockdown cells and negative control cells were maintained with 600 μg/ml G418 (Sigma).

The cDNA of full-length human MUC1 (22 TR) was kindly provided by Dr O.J. Finn at the University of Pittsburgh (Pittsburgh, PA, USA) and was inserted into a lentiviral vector pWPXLd, in which the GFP sequence was substituted by MUC1. The integrity of the constructs was confirmed by sequencing (Genewiz). A virus packaging cell line, HEK293T, was seeded at 1 × 10^6^ cells per 100-mm dish. Twenty-four hours later, the cells were subjected to transfection for 4 h with Lipofectamine (Invitrogen) and 3.0 μg of the pWPXLd-MUC1 overexpression construct (or pWPXLd empty vector) with the PAX2 and PMD2.G packaging vectors. After culturing the cells for 48 h, the filtered culture supernatants were used for infection. The Bel7402 and Hep3B HCC cell lines were infected with a viral solution carrying MUC1 in the presence of 5 μg/ml polyblene (Sigma) for 6 h and then were incubated in 10% FBS/IMDM. Two MUC1-overexpressing HCC cell lines (7402-MUC1 and Hep3B-MUC1) and two negative controls (7402-EV and Hep3B-EV) were identified by flow cytometry and Western blotting.

### Flow cytometry

To analyze MUC1 expression, cells (1 × 10^6^) were fixed with paraformaldehyde for 1 h and washed twice with a fluorescence-activated cell sorter (FACS) solution (PBS containing 2% FCS and 0.1% NaN_3_). Subsequently, the cells were incubated with a mouse monoclonal antibody against MUC1 tandem repeats (GP1.4, Neo Markers) on ice for 30 min, washed twice with FACS solution and stained with PE-conjugated goat anti-mouse secondary antibody (Proteintech Group, Chicago, IL, USA) at a dilution of 1:100 for 30 min on ice in the dark. After washing twice with FACS solution, the expression of MUC1 was analyzed by flow cytometry (FACSCalibur; BD Biosciences). The results are representative of three independent experiments.

### Quantitative real-time PCR (qRT-PCR)

After the cells were harvested, RNA was isolated using TRIzol (Invitrogen Life Technologies). Total RNA was converted to cDNA using M-MLV reverse transcriptase and oligo (dT) primers (Promega Corporation, Madison, WI, USA) according to the manufacturer's protocol. Reverse transcribed products were used to amplify MUC1, Smad3 and TβRI by qRT-PCR. qRT-PCR was performed using a FastStart Universal SYBR Green Master (Rox) (Roche) on an ABI7300 instrument. The primer sequences used for qRT-PCR were 5′-TCTCCAATGTCAACAGGAATGC-3′ (forward) and 5′-GAGCCGCACGCCTCTTC-3′ (reverse) for Smad3, and 5′-AGTTGCGTTACACCCTTTC-3′ (forward) and 5′-CCTTCACCGTTCCAGTTT-3′ (reverse) for β-actin. The expression of each investigated gene was normalized to the housekeeping gene β-actin. Data were calculated using the 2^−ΔΔ*C*T^ method and are presented as the fold change in gene expression relative to the negative control sample, from three independent experiments. For the negative control sample, 2^−ΔΔ*C*T^ equals 1.

### Cell viability assay

Cell viability was determined at different time points using a WST-1 cell viability assay according to the manufacturer's protocol (Roche Diagnostics, Mannheim, Germany). Triplicate wells containing 5 × 10^3^ cells were evaluated for viability. The absorbance was measured using a microplate reader at a wavelength of 450 nm (BioTek Instruments, Inc., Winooski, VT, USA). The relative cell viability was calculated as the A450 nm (MUC1-silenced or overexpressed cells at Tn)/A450 nm (control at Tn) × 100%. VT, USA), from three independent experiments.

### Immunofluorescence confocal microscopy assay

Cells were fixed with 4% paraformaldehyde and permeabilized with 0.2% Triton X-100. After blocking with 2% bovine serum albumin (BSA), the cells were incubated with hamster anti-MUC1 antibody (Ab-5; Neomarker) and mouse anti-JNK antibody (Santa Cruz) overnight at 4°C. After washing, FITC-conjugated goat anti-hamster (Santa Cruz), and PE-conjugated goat anti-mouse IgG (Proteintech Group, Chicago, IL, USA) were added for 1 h at 37°C in the dark. The nuclei were stained with 1 μg/ml 4′,6-diamidino-2-phenylindole (DAPI) (Sigma-Aldrich, St. Louis, MO, USA) for 10 min, and the cells were observed by confocal laser scanning microscopy. The confocal laser scanning microscopy was carried out using an Olympus FV1000 microscope equipped with a multiline argon laser, 405, 488, 543 nm, and 30 mW laser class 3D laser.

### Western blotting and co-immunoprecipitation analysis

Cells were lysed with RIPA lysis buffer, and the protein concentrations in the cell lysates were measured using a BCA protein assay kit (Beyotime Biotechnology, Jiangsu, China). Equal amounts of cell lysate protein were separated by 10% SDS-PAGE and transferred to PVDF membranes (Millipore, Billerica, MA, USA). Primary antibodies against the following were used: MUC1 (GP1.4) (1:2000; NeoMarkers), c-Myc (1:1000), GAPDH (1:1000; both from Epitomics, Burlingame, CA, USA), p21^WAF1^ (1:1000), JNK (1:1000), p-JNK (1:1000; all from Cell Signaling Technology), Smad3 (1:1000), p-Smad3L (Ser-213), and p-Smad3C (Ser-423/425) (1:1000; all from Abcam). For co-immunoprecipitation analysis, the cell lysates were pre-cleared with protein G agarose beads (Beyotime Biotechnology, Jiangsu, China) for 3 h at 4°C. Then, equal amounts of sample lysate were incubated with either 1.0 μg of mouse IgG or anti-JNK antibody (Santa Cruz Biotechnology) for 16 h at 4°C, followed by precipitation with protein G agarose beads. Immunoprecipitated proteins from cell lysates and total cell lysates were subjected to immunoblot analysis with anti-MUC1-CT (Ab-5; Neomarker). The signals from the Western blotting were normalized to the constitutive GAPDH control. The results are representative of three independent experiments.

### *In vivo* tumor growth and immunohistochemical staining assays

BALB/c nude mice (4–6 weeks old) were purchased from Beijing HFK Bioscience Co., Ltd., China. Animals were maintained in specific pathogen-free conditions and environment under controlled conditions of light and humidity. Animal experiments were conducted in accordance with the National Institutes of Health Guide for the Care and Use of Laboratory. The mice were randomly divided into 4 groups (5 animals/group) that were designated as the SMMC-7721 group, the NC group, the MR1-D4 group and the MR1-D9 group. Cells (2 × 10^6^) were subcutaneously injected into the right flank of each mouse. On day 21 post-injection, the tumors were dissected, fixed in 10% neutral-buffered formalin and embedded in paraffin for immunohistochemical staining using antibodies against MUC1 (GP1.4), p-JNK, p-Smad3L (Ser-213), c-Myc, and an UltraSensitiveTM SP (Mouse/Rabbit) IHC Kit (MaiXin.BIO., Fuzhou, China). Liver tissues from hemangioma patients and HCC patients were used similarly to the mouse tumors. The sections were examined on an inverted fluorescence microscope (IX71; Olympus).

### Statistical analysis

Data were expressed as the means ± SD. SPSS 17.0 software was used for analysis. All experiments (do not include the nude mice subcutaneous transplant tumor model experiment) were repeated at least three times. The statistical significance of a difference between two groups was assessed using Student's *t*-tests, and *P* < 0.05 was considered to indicate a statistically significant result.
